# Inter-organismal signaling and management of the phytomicrobiome

**DOI:** 10.3389/fpls.2015.00722

**Published:** 2015-09-14

**Authors:** Donald L. Smith, Dana Praslickova, Gayathri Ilangumaran

**Affiliations:** Plant Science Department, McGill University/Macdonald Campus, Sainte Anne de Bellevue, QC, Canada

**Keywords:** inter-organismal signals, phytomicrobiome, plant agriculture, crop productivity, plant stress

## Abstract

The organisms of the phytomicrobiome use signal compounds to regulate aspects of each other’s behavior. Legumes use signals (flavonoids) to regulate rhizobial *nod* gene expression during establishment of the legume-rhizobia N_2_-fixation symbiosis. Lipochitooligosaccharides (LCOs) produced by rhizobia act as return signals to the host plant and are recognized by specific lysine motif receptor like kinases, which triggers a signal cascade leading to nodulation of legume roots. LCOs also enhance plant growth, particularly when plants are stressed. Chitooligosaccharides activate plant immune responses, providing enhanced resistance against diseases. Co-inoculation of rhizobia with other plant growth promoting rhizobacteria (PGPR) can improve nodulation and crop growth. PGPR also alleviate plant stress by secreting signal compounds including phytohormones and antibiotics. Thuricin 17, a small bacteriocin produced by a phytomicrobiome member promotes plant growth. Lumichrome synthesized by soil rhizobacteria function as stress-sensing cues. Inter-organismal signaling can be used to manage/engineer the phytomicrobiome to enhance crop productivity, particularly in the face of stress. Stressful conditions are likely to become more frequent and more severe because of climate change.

## Background

The perspectives provided in this theme volume illustrate that members of the phytomicrobiome utilize inter-organismal signal compounds to affect the behavior of the plants they associate with, and signal compounds from the plants regulate the behavior of the phytomicrobiome. Presumably, one organism alters the behavior of another for its own benefit, but often to the benefit of the other organism as well, leading to mutualistic symbiosis. An example of this is improved stress tolerance in a plant by a signal compound from an associated microbe, where the resulting enhanced plant growth means expanded niche space and more reduced carbon for the specific phytomicrobiome member.

## Signaling in the Legume-rhizobia Symbiosis

Plants must allow beneficial microorganisms to colonize near them or in their tissues in order to establish mutualistic relationships. This kind of close association (for example, the legume-rhizobia symbiosis, where rhizobia reside inside the legume roots) necessitates a filtering system in the plants, disallowing unsuitable microorganisms, perhaps pathogens that could harm their tissues. On the other side, a microbe entering a disadvantageous plant would risk being recognized as unacceptable and killed. Signal/recognition compounds facilitate communication between mutually beneficial organisms and ensure continuum of their relationship until senescence. Flavonoids (examples: luteolin, 7,4′ dihydroxyflavone, quercetin, kaempferol, myricetin, genistin, etc.,) in the rhizosphere are constituents of root exudates and well studied for their function as legume-to-rhizobia signal compounds (Nelson and Sadowsky, 2015). Their structural diversity and substitutions in the carbon skeleton determines their characteristic function (Weston and Mathesius, 2013). The release of specific flavonoids (or mixtures) from a legume host is only recognized by certain rhizobial species, which partially determines the host-symbiont specificity. The flavonoids diffuse through the rhizobial membrane and bind to NodD proteins in rhizobia, which then activate transcription of *Nod* genes involved in synthesis of nodulation factors (NF; Hassan and Mathesius, 2012). Altered flavonoid profiles at different symbiosis stages regulate Nod factor synthesis (Dakora et al., 1993). Flavonoids also cause auxin accumulation in root tissues that initiates nodule formation and differentiation (Hassan and Mathesius, 2012). Flavonoids regulate development of nodules and phytoalexin resistance in rhizobia (Cooper, 2004). Thus, these signal compounds regulate the behavior of appropriate partner organisms down to the gene expression level.

A range of very diverse non-flavonoid compounds present in the root exudates also induces *Nod* genes in some rhizobia (Mabood et al., 2014): betaines (stachydrine and trigonelline; Cooper, 2007), aldonic acids (erythronic and tetronic acids), and jasmonates (jasmonate and methyl jasmonate; Mabood et al., 2006). The jasmonates have been commercialized and products are now available (http://agproducts.basf.us/products/vault-hp-plus-integral-for-soybeans-inoculant.html).

Activated rhizobial *Nod*-genes secrete signals (Nod factors) back to the plant: lipochitooligosaccharides (LCOs) and exopolysaccharides (EPS). LCOs are conserved at the core but are diverse due to degree of saturation and the substitutions (glycosylation or sulfation) in the N-Acetyl chain at both reducing ends and vary widely between different rhizobial species, which are essential for host plant specificity (Oldroyd, 2013). Genes at the loci of Nod factors perception encode receptor like kinases with N-Acetyl glucosamine binding lysine motifs (LsyM RLK), which include Nod factor receptors (NFR1), NFR5, LysM receptor kinase 3 (LYK3), Nod factor perception (NFP). NFR/NFP binds to NF and are essential in determining NF specificity of rhizobial symbionts and activation of nodulation signaling (Oldroyd, 2013). Signaling from the receptor complex generates calcium oscillations in the nucleus of cortical cells, which activate a localized protein, calcium and calmodulin (CaM) dependent serine/threonine protein kinase (CCamK), and phosphorylates CYCLOPS, which is required for rhizobial colonization and nodule development (Oldroyd, 2013). The rhizobial specific gene expression is regulated by the Nodulation signaling pathway (*NSP1* and *NSP2*) and encodes GRAS domain transcription factors involved in nodulation specific functions. They are associated with promoters of Nodulation inception genes (*NIN*) and early nodulation genes (*ENOD*) and ensure that nodulation is stimulated under appropriate circumstances (Kalo et al., 2005; Smit et al., 2005).

In some rhizobia-legume systems (for example, *Bradyrhizobium*, and *Glycine soja*) application of correct Nod factors (LCOs isolated from *B. elkanii*) trigger formation of complete and anatomically precise, albeit, empty nodules (Stokkermans and Peters, 1994). It is impressive that the external application of a signal compound can lead to complete organogenesis.

Although many parallels are observed in the signaling mechanisms, plants exhibit subtle regulatory pathways to establish mutualistic associations and protect from pathogenesis (Toth and Stacey, 2015). During rhizobial infection, legume defense responses are elicited in the early stages but suppressed soon after (Libault et al., 2010). Increased activation of mitogen activated protein kinase (MAPK) and production of reactive oxygen species were observed in legumes when inoculated with rhizobia (Jamet et al., 2007; Lopez-Gomez et al., 2012). Chitooligosaccharides, chitosan, lipopolysaccharides, and peptidoglycan associated with fungal and bacterial pathogens are recognized as microbe-associated molecular patterns (MAMPs) by pattern recognition receptors (PRRs) in the plant cell membrane (Dangl and Jones, 2001; Zipfel, 2014). Recognition of MAMPs is crucial for the activation of MAMP triggered immunity (MTI) in plants, which triggers expression of defense related genes, leading to structural hardening (callose formation) of plant tissues, accumulation of phytoalexins and antimicrobial peptides (Ahuja et al., 2012). NF remain active even after nodulation, suggesting a role in suppression of MTI (Liang et al., 2013). Exopolysaccharide of rhizobia (example succinoglycan from *Sinorhizobium meliloti*) is known to supress plant immunity (Aslam et al., 2008). LCO recognition has been evolved from a pathogenic role to symbiosis. Even though LCOs are structurally similar to chitin oligomers (MAMPs) and their recognition is mediated by LysM RLK, modifications in amino acid sequences of LysM RLK which confer specificity to recognition of LCO or chitin oligosaccharides (Nakagawa et al., 2011). For example, chimeric proteins in the ectodomain of chitin elicitor receptor kinase (CERK1) for chitin perception are replaced with ectodomain of NFR involved in NF recognition (Zhang et al., 2007).

Effector proteins secreted by pathogens trigger effector mediated immunity (ETI) in plants due to activation of resistance (R) genes encoding nucleotide-binding site—leucine rich repeat proteins (Jones and Dangl, 2006). Leucine rich repeats receptor like kinases (LRR—RLK) are involved in NF perception and nodule formation (Endre et al., 2002). Effectors are transported and injected into the host cytoplasm through type III (T3SS) and type IV (T4SS) secretion systems. Effector proteins of rhizobia (NopM of *S. fredii* NGR234, NopL from *S. fredii* USDA247) have been shown to facilitate colonization of rhizobia in roots, prevent MAPK signaling, supress the plant immune system, affect formation of nitrogen-fixing nodules, timing of nodule establishment and final number of nodules formed (Zhang et al., 2011). Interestingly, rhizobial NF, T3SS and T4SS depend on a common regulator activated by legume secreted flavonoids (Gourion et al., 2015).

Bacteroid differentiation inside the nodule is regulated by antimicrobial peptides (nodule cysteine rich peptides), which functions similar to plant defensins (de Velde et al., 2010). The bacteroids are separated from the host by a symbiosome membrane and immune activity is modulated inside the nodules and the expression of defense related genes is relatively low (Limpens et al., 2013). The plant controls the duration of symbiosis and regulates the senescence of nodules and the suppression of plant immunity reverses during nodule senescence (Puppo et al., 2005). The number of nodules is controlled by the legumes through a process called autoregulation of nodules (AON; Mortier et al., 2012). Shoot derived signals involve production of cytokinins and downstream signaling to the roots regulates AON (Sasaki et al., 2014).

Rhizobia signaling and associations can be affected by other members of the phytomicrobiome, this is because they function together as a consortia exerting synergism, playing a vital role in plant growth, nutrient uptake, alleviation of abiotic stress, and protecting from disease. The more frequently studied co-inoculation partners of rhizobia are *Bacillus* species. Inoculation of *Rhizobium* with *Bacillus* strains improved root structure and nodule formation in bean, pigeon pea and soybean (Halverson and Handelsman, 1991; Petersen et al., 1996; Srinivasan et al., 1997; Rajendran et al., 2008). Inoculation of pea with *Bacillus simplex* 30N-5 and *Rhizobium leguminosarum* bv. *viciae* 128C53 increased root nodulation and plant growth (Schwartz et al., 2013). When pea plants carrying *DR5*::*GUS* promoter are co-inoculated with *B. simplex* 30N-5 and *R. leguminosarum* bv. *viciae* expression of GUS was higher in nodule meristems and young vascular bundles of developing nodules (Schwartz et al., 2013). *Azospirillum brasilense* co-inoculated with *R. tropici* on bean relieved negative effects of salt stress on *nod* genes transcription (Dardanelli et al., 2008). Co-inoculation of rhizobia and arbuscular mycorrhizal fungi (AMF) promoted growth of soybean under low phosphorous and nitrogen conditions, indicated by increase in shoot dry weight (Wang et al., 2011).

The legume-rhizobia symbiotic relationship tends to be less specific in tropical agriculture, involving much wider sets of rhizobial partners, while it is often quite specific in the temperate zones (Dakora, 2000). A wider range of rhizobia forming relationships with any given legume, and the more diverse signaling involved, may alter the effect of environmental conditions on the nitrogen-fixing symbiosis for that particular legume species. Exploitation of the rhizobia-legume symbiosis has occurred for over a century yet, there is considerable scope for improved understanding of this complex relationship in tropical zones.

## Other Phytomicrobiome Signaling Systems

While the legume-*Rhizobium* symbiosis is well understood of signaling interactions, given its significance of biological nitrogen fixation, extensive research in other phytomicrobiome signaling systems has been conducted. Mycorrhizal symbiosis uses a signaling system similar to that of the legume-rhizobia symbiosis (Harrison, 2005; Oldroyd, 2013) and it plays a critical role in solubilisation of minerals and plant protection. In this association plants emit strigolactones, triggering production of Myc factors including LCOs by the fungus and stimulate hyphal branching (Bonfante and Requena, 2011). AMF have a broad host range and hence they produce diverse array of LCOs for recognition by the host plants. LysM RLK are also associated with mycorrhizal colonization (Young et al., 2011). It would be interesting to study the mechanisms employed by the plants to differentially recognize mycorrhizal and rhizobial LCOs.

Nitrogen fixing symbiotic association occurs between *Frankia* and actinorhizal plants. *Frankia* sp. colonizes roots of actinorhizal plants and induces root hair curling and nodule formation similar to those observed in legumes suggesting common symbiotic mechanisms but with important structural differences, particularly the signaling compounds produced by *Frankia* differ from rhizobia (Pawlowski and Bisseling, 1996; Gherbi et al., 2008).

Many plant growth promoting rhizobacteria (PGPR; example, *Bacillus*, *Pseudomonas*, *Serratia*, *Azospirillum*, *Acetobacter*, etc.,) secrete phytohormones, such as cytokinins, gibberellins, auxin, and ACC deaminase and influence plant growth and functions (Vessey, 2003). They are also capable of alleviating drought stress by promoting root growth and hampering stomatal conductance (Vessey, 2003; Gray and Smith, 2005). The phytomicrobiome also improves the uptake of nutrients by forming siderophores or solubilizing phosphates and other minerals (Vessey, 2003). Phytomicrobiome members synthesize and excrete a range of inter-organismal signal compounds that defend their host plant against pathogens and abiotic stresses: broad-spectrum antibiotics, lytic enzymes, organic acids and other metabolites, proteinaceous exotoxins and antimicrobial peptides (bacteriocins).

Several products of PGPR have been commercialized as biofertilizers and biocontrol agents owing to their diverse modes of action. There is considerable scope for application of phytomicrobiome signals in agriculture. For instance, *Bacillus thuringiensis* NEB17 produces the bacteriocin thuricin 17. Intriguingly, this peptide is also a bacteria-to-plant signal that stimulates the growth of many plants (Lee et al., 2009). Thuricin 17 (10^–9^ to 10^–11^ M) changes the hormone levels of *Arabidopsis* and soybean (increased IAA and SA) and causes profound alterations in the proteome (major increases in energy related proteins; Subramanian, 2014). Thuricin 17 almost completely overcomes the negative plant growth effects of salt stress (250 mM NaCl). For the producer bacterium *B. thuringiensis* NEB 17, thuricin 17 is a dual function peptide, acting both as a bacteriocin that reduces competition from closely related bacteria, and to enlarge the available niche space by promoting plant growth. *Bacillus subtilis* OKB105 contains genes (*yecA*, *speB*, *ACO1*) involved in synthesis of spermidine, a plant growth stimulating polyamine (Xie et al., 2014).

Bacterially produced lumichrome (breakdown product of riboflavin) accelerates leaf production, onset of stem elongation, and leaf area development (at a concentration of 5 × 10^–9^ M), leading to greater production of biomass in many plants (maize, sorghum, tomato, lotus), related to enhanced starch and ethylene metabolism. Adversely, 10-fold greater concentrations can retard plant growth and development (Matiru and Dakora, 2005; Gouws et al., 2012). Similar effects were observed in legumes (soybean, cowpea) in response to the signal compounds (lipopolysaccharides and lumichrome), suggesting their role in the nitrogen-fixing symbiosis. Lumichrome promotes nodulation and mycorrhizal establishment in legumes (Dakora and Phillips, 2002). Lumichrome also helps plants deal with drought and salinity stress (Kanu and Dakora, 2009).

Quorum sensing signals including those of beneficial bacterial such as rhizobia (Zarkani et al., 2013) can elicit immune responses (Schenk et al., 2012; Hartmann et al., 2014), and change hormone profiles in plants, inducing those regulating growth responses and disease resistance (Hartmann and Schikora, 2012). Quorum sensing regulates mobility, virulence and biofilm formation in bacteria. Biofilm formation (bacteria embedded in a thick matrix of EPS, proteins and water) enables bacteria to adhere to host tissues. Biofilm improves plant growth, root proliferation (*Azospirillum* in wheat) and function in as biocontrol (*B. subtilis*, Farrar et al., 2014). In the case of *N*-acyl-homoserine lactones (AHL), the length of the lipid side chain dictates characteristics of the signal compound’s activity (Schikora et al., 2011). Quorum sensing in the phytomicrobiome will be the subject of an upcoming Frontiers in Plant Science theme volume (Plant responses to bacterial quorum sensing signal molecules, topic editors Schikora A and Hartmann A).

## Engineering the Phytomicrobiome

Given our intense reliance on higher plants for food and other resources, our expanding understanding of the phytomicrobiome associated with these plants, advances in genetic engineering and synthetic biology, it seems reasonable to consider “engineering” the phytomicrobiome to improve crop productivity, including enhancement of photosynthesis and growth, nutrient assimilation, disease and insect resistance and improved ability to resist increases in abiotic stresses likely to be associated with environmental disturbances, or even mitigating the impact of climate change through CO_2_ sequestration. The host plant with its phytomicrobiome constitutes a holobiont (Hartmann et al., 2014), a collective community with broader genomic, proteomic, metabolomics and physiologic capacity, making it better able to adjust to environmental (biotic and abiotic) challenges. The potential to alter the composition of the microbial consortia residing near, on or in plant tissues has been explored through inoculation processes to some extent. The inoculation strategy to manipulate the microbiome focuses on co-inoculation of several strains of PGPR, arbuscular mycorrhizal fungi and other endosymbionts. Increase in the abundance of beneficial microbes in the rhizosphere (for example biofertilizers) has resulted in less disease incidence and high levels of microbial activity (Bunemann et al., 2006).

Understanding plant microbe interactions requires a holistic approach to analyze this complex and dynamic system. However, the difficulty to readily culture many members of phytomicrobiome (for example, obligate endosymbionts) in the laboratory can be overcome by culture independent techniques such as metagenomics, metaproteomics, and metabolomics and usage of next generation sequencing tools to understand the complexity of the phytomicrobiome (Bulgarelli et al., 2012; Quiza et al., 2015). Our ability to implement large-scale manipulations of the microbial populations is currently limited. Plant microbiome engineering facilitates modulation of nutrient cycling, synthesis of phytohormones, production of antibiotics (biocontrol agents), leading to improved plant growth and resistance to disease, insects, drought, salinity stress, etc. (Quiza et al., 2015). Introducing recombinant strains in the rhizosphere could improve the persistence of endogenous microbial population by horizontal gene transfer (Taghavi et al., 2005) and community level microbiome engineering could result in higher resilience across disruptive environments (Loreau et al., 2001). The ability to engineer the phytomicrobiome will be pivotal in furthering long-term sustainability of agricultural crop production and affecting related issues such as climate change, human health and global food security (Quiza et al., 2015). While we are progressing in our understanding of mechanisms involved in the interspecies interactions, nature of the complex relationships within the phytomicrobiome, role of the host plants and its microbiome as a holobiont (Lakshmanan et al., 2014), engineering the whole metaorganism is a promising strategy that finds application in nitrogen fixation, disease control, nutrient cycling and phytoremediation (Bakker et al., 2013; Bell et al., 2014).

It is clear that members of the phytomicrobiome exchange signal compounds that are effective at hormonal concentrations, so that inter-organismal, indeed, inter-kingdom exohormones are now understood to play a crucial role in controlling the growth, composition and development of plants, including the crop plants that we depend on as food sources. The commercial deployment of LCOs in non-legume crop plants (Souleimanov and Prithiviraj, 2002; Prithiviraj et al., 2003) indicates that there is enormous scope for application of these compounds, to help crop plants be more productive, and to remain productive under the more challenging environmental conditions of climate change. Indeed, many of the positive effects of phytomicrobiome signals on plant growth seem to involve activation of stress response systems. Understanding the mechanisms and consequences of signal interactions occurring between the phytomicrobiome and host plants and development of methods to manipulate these interactions for increased plant growth, is an important challenge for this century.

### Conflict of Interest Statement

The DS conducts research in collaboration with Inocucor Technologies, which manufactures and sells microbial consortia for inoculation on plants, where the research is funded through a Canadian Federal Government program (Mitacs) which levers industrial funding. The other authors declare that the research was conducted in the absence of any commercial or financial relationships that could be construed as a potential conflict of interest.
